# Supplementary material for: Effectiveness of ATMAN psychological intervention in reducing self-harm in young people in India: a mixed method case series
Source: Glob Ment Health (Camb). 2025 Mar 14;12:e41. doi: 10.1017/gmh.2025.26 (PMC12037354; doi:10.1017/gmh.2025.26)
Supplement: Aggarwal et al. supplementary material [file S2054425125000263sup001.docx]

**Supplementary Table 1. List of modules and elements of ATMAN intervention**

| Modules | Elements | Sessions |
| --- | --- | --- |
| Crisis Management | Psychoeducation (including case conceptualisation), crisis plan (including chain analysis), distraction strategies, | Session 1 |
| Problem solving | Problem solving | Session 2 |
| Emotion regulation | Emotion regulation, self-soothing | Session 3-4 |
| Social network strengthening | Mobilizing social support, family education | Session 4-5 |
| Optional modules- additional session/s 1-3 | |  |
| Family module | Family communication | To be used in case there is a significant breakdown in family communication |
| Substance use | Motivational enhancement | To be used when substance use is a significant problem |
| Assertiveness training | Assertiveness skills | Can be used in younger population (adolescents) |

**Supplementary Table 2. Characteristics of ATMAN Treatment Completers versus Non-Completers**

| Gender | Treatment completers (n-18) | Treatment non-completers (n-9) |
| --- | --- | --- |
| Female (%) | 13 (72%) | 5 (56%) |
| Age in years, mean (SD) | 18.9 (3.3) | 19.0 (4.3) |
| Mental health diagnoses |  |  |
| Serious mental illness (schizophrenia, bipolar disorder) | 3 (17%) | 2 (22%) |
| Depressive and/or anxiety disorders | 10 (56%) | 4 (44%) |
| Personality disorders/ traits | 5 (28%) | 2 (22%) |
| Substance use, neurodevelopmental disorders | 3 ( 17%) | 1 (11%) |
| Medications |  |  |
| Antidepressants | 14 (78%) | 4 (44%)***** |
| Antipsychotics (oral/ depot) | 5 (28%) | 4 (44%)* |
| BSI, mean (SD) | 22.7 (7.1) | 14.9 (4.6) |
| PHQ-9, mean (SD) | 15.5 (7.5) | 16.8 (7.1) |

BSI Beck’s Suicidal Ideation Scale; PHQ Patient Health Questionnaire; * p<0.05,

SD standard deviation

**Supplementary Table 3. Paired t-tests with ATMAN pre-, and post-therapy time points (n-18)**

| Scales | Pre-therapy | | Post- therapy | | Difference* (95% CI) | | t-statistic | | p value | |
| --- | --- | --- | --- | --- | --- | --- | --- | --- | --- | --- |
|  | **Mean (SD)** | **Mean (SD)** | |  | |  | |  | |  |
| BSI | 22.7 (7.1) | 8.6 (5.3) | | 14.0 (10.7, 17.4) | | -8.74 | | <0.001 | |  |
| PHQ-9 | 15.5 (7.5) | 5.9 (4.6) | | 9.6 (6.2, 13.1) | | -5.88 | | <0.001 | |  |

**Difference calculated as Pre therapy-Post therapy*

**Supplementary Table 4. BSI and PHQ-9 scores at baseline, post-intervention and 10-months follow-up for specialists and non-specialist providers**

| **BSI** | Baseline | Post intervention | At 10 months Follow-up |
| --- | --- | --- | --- |
| **Specialist** |  | | |
| **BSI, mean (SD)** | 22.8 (6.2) | 6.6 (5.2) | 6.1 (3.9) |
| **BSI, median (IQR)** | 22.5 (20.0, 27.0) | 4.5 (3.0, 13.0) | 4.5 (3.5, 8.0) |
| **Non-Specialist** |  |  |  |
| **BSI, mean (SD)** | 22.5 (8.7) | 11.1 (4.5) | 4.4 (0.5) |
| **BSI, median (IQR)** | 24.5 (13.0, 28.0) | 11.5 (8.5, 14.5) | 4.0 (4.0, 5.0) |
| **PHQ-9** |  |  |  |
| **Specialist** |  | | |
| **PHQ, mean (SD)** | 18.7 (5.6) | 5.6 (4.8) | 4.6 (2.7) |
| **PHQ, median (IQR)** | 19.0 (16.0, 23.0) | 3.5 (2.0, 9.0) | 3.5 (2.5, 7.0) |
| **Non-Specialist** |  | | |
| **PHQ, mean (SD)** | 11.5 (8.0) | 6.3 (4.7) | 4.4 (3.2) |
| **PHQ, median (IQR)** | 11.5 (6.0, 17.0) | 5.0 (2.0, 11.5) | 4.0 (3.0, 7.0) |

**Supplementary Table 5. Interview Guides for exit interviews and follow-up interviews**

**Exit interview questions**

1. What drove you to participate in the project? What were your expectations out of it?
2. Were you able to clearly understand the nature of the project?
3. Did you experience any difficulties during the recruitment or intervention process? `
4. How did you use the intervention elements?
5. How did you find involving your parents/ family members in the process?
6. What do you think about the support you received during your participation in ATMAN and your prior experiences of getting help?,
7. What did you find the most difficult about the process?
8. Did you experience any challenges while getting the treatment and associated research activities?

**Follow-up questions**

1. How have you been since finishing ATMAN intervention?
2. Did you use any of the strategies learnt during the intervention?
3. How did you use the intervention elements ?
4. Did you experience any challenges while using the intervention elements?
5. How did you find involving your parents/ family members in the process?
6. What do you think about the support you received during your participation in ATMAN and your prior experiences of getting help?
7. Any suggestions about making the intervention more useful.
